# Early versus delayed cord clamping in small for gestational age infants and iron stores at 3 months of age - a randomized controlled trial

**DOI:** 10.1186/s12887-018-1214-8

**Published:** 2018-07-18

**Authors:** Abhishek Chopra, Anup Thakur, Pankaj Garg, Neelam Kler, Kanwal Gujral

**Affiliations:** 1 Department of Neonatology, Sir Ganga Ram Hospital, New Delhi, India; 2 Department of Obstetrics and Gynecology, Sir Ganga Ram Hospital, New Delhi, India

**Keywords:** Delayed cord clamping, Early cord clamping, Small for gestational age, Ferritin, Hemoglobin, Polycythemia, Partial exchange

## Abstract

**Background:**

Delayed cord clamping is the standard of care in infants not requiring resuscitation; however effects of cord clamping strategies have not been evaluated systematically in small for gestational age (SGA) infants. The primary objective was to compare effects of delayed cord clamping (DCC) and early cord clamping (ECC) on serum ferritin at 3 months in SGA infants born at ≥35 weeks. The secondary objectives were to compare hematological parameters, clinical outcomes in neonatal period and growth at 3 months of age.

**Methods:**

All eligible infants with fetal growth restriction were randomized to two groups, DCC at 60 s or ECC group in which the cord was clamped immediately after birth.

**Results:**

Total of 142 infants underwent randomization and subsequently 113 infants underwent definite inclusion. At 3 months, the median (IQR) serum ferritin levels were higher in DCC group, compared to ECC; 86 ng/ml (43.35–134.75) vs 50.5 ng/ml (29.5–83.5), *p* = 0.01. Fewer infants had iron deficiency in DCC group compared to ECC group; 9 (23.6%) vs 21 (47.7%), *p* = 0.03 [NNT being 4; 95% CI (2–25)].The proportion of infants with polycythemia was significantly higher in DCC group; 23 (41.81) % vs 12 (20.6%), p = 0.01. There was no difference in proportion of infants with symptomatic polycythemia or those who underwent partial exchange transfusions. Clinical outcomes and mortality were similar.

**Conclusions:**

DCC improves iron stores in SGA infants ≥35 weeks at 3 months of age without increasing the risk of symptomatic polycythemia, need for partial exchange transfusions or morbidities associated with polycythemia.

**Trial registration:**

Our trial was retrospectively registered on 29th May 2015 through Clinical trials registry India. Registration number: CTRI 2015/05/005828.

## Background

The umbilical cord acts as a conduit for gas exchange, nourishment and endocrinal homeostasis of the fetus. Umbilical cord clamping signifies a landmark period to transform the fetus to an independent entity. Two commonly practiced techniques of cord clamping are – “early cord clamping” (ECC) (clamping in less than 1 min after birth) and “delayed cord clamping” (DCC) (clamping at 1 to 3 min of birth) [1].

DCC has become a standard of care in infants not requiring resuscitation [1–4]. DCC provides 25–35 ml of blood per kg in term infant (equivalent to 1–3 months of infant’s iron requirements) and improves iron stores leading to reduction in iron deficiency [5, 6]. Iron deficiency during the fetal or postnatal period can alter brain structure, neurochemistry and cognitive function. This can lead to long term cognitive and motor impairment, which cannot be corrected by iron supplementation later in life [7].

Small for gestational age (SGA) infants are those with birth weight less than 10th centile for the gestation and sex. Studies have shown that these infants have low iron stores at birth and are at risk of developing iron deficiency later [8, 9]. This is due to placental vascular insufficiency mediated impairment in iron transport and increased utilization of iron to meet chronic hypoxia induced erythropoiesis. ECC would deprive them of additional placental blood, further accentuating the risk of iron deficiency. However these infants have an inherent risk of polycythemia and its related complications, which may be aggravated by DCC. Cord clamping strategies in SGA infants and their clinical impact has not been evaluated systematically in randomized controlled trials. We conducted this study to compare the effect of two cord clamping strategies on early neonatal outcomes and iron stores at 3 months of age.

## Methods

We conducted a parallel randomized controlled trial with 1:1 randomization comparing early and delayed cord clamping. The study was conducted from November 2013 to February 2015 at Sir Ganga Ram Hospital, a tertiary care center in northern India. Our trial was retrospectively registered on 29th May 2015 through Clinical trials registry India. Registration number: CTRI 2015/05/005828.

All pregnant women who underwent antenatal ultrasonography (USG) from second trimester onwards were screened. Gestational age was derived from the first trimester ultrasound if available or from last menstrual period The fetal weight was estimated and plotted on Hadlock chart [10]. They were eligible if there was evidence of fetal growth restriction (weight for gestation less than 10th centile) on antenatal ultrasonography. Unique identification numbers of eligible pregnant women were entered in a dedicated software. On admission to the hospital for safe confinement, an automated message was sent to the principal investigator who tracked them for further eligibility. Exclusion criteria were infants born to mothers with placental abruption or previa, those with antenatally diagnosed major congenital malformations, Rh isoimmunised and multiple pregnancies. Post randomization exclusion criteria were infants born at ≥10th centile and those needing resuscitation. Pregnant women were randomized to early or delayed cord clamping groups if gestational age at delivery was ≥35 weeks. A written informed consent for participation in the study was obtained from either parent prior to delivery. Training sessions were held prior to study initiation to apprise the research and resuscitation team of study protocol and timing of events. These training sessions were re-enforced every 3 months.

A computer based variable block (block size 4 and 6) random sequence was generated by an independent researcher using random number table. This allocation sequence was concealed in sealed opaque envelopes. The opaque envelopes were sequentially numbered by an independent staff member and were kept in the Neonatal Intensive Care Unit (NICU). All deliveries were attended by neonatology fellow or consultant on call. Prior to delivery, the attending neonatologist called the NICU helpline and contacted the nurse in charge, who opened the sealed opaque envelope and disclosed the intervention (DCC/ECC). The nature of intervention prevented us from blinding.

In ECC group, the obstetrician clamped the cord immediately after birth. In the DCC group, cord was clamped after 60 s. Stopwatch was started when infant’s buttocks (or head if breech) were delivered from the vagina (or the uterus in cesarean section). To facilitate DCC, the time elapsed was counted aloud by the attending neonatologist in 10 s interval. The exact time of cord clamping was noted in both the groups. During this time, the infant was held in linen at the level of introitus in vaginal delivery or on the legs in cesarean section. Care was taken not to apply traction on the cord. Milking of the cord was not done. After the cord was clamped, infant was cared by the attending neonatologist as per NRP 2010 guidelines [4]. Birth weight was entered in Fenton gestation and sex specific online calculator 2013 [11] and infant was classified as SGA if the birth weight was less than 10th centile [12]. Infants whose birth weight was at or above 10th centile and those needing resuscitation were excluded from the study. In both ECC and DCC groups, clinical care was provided as per the standard unit protocol.

All blood samples were drawn from a large peripheral vein. At 2 h of life hemoglobin and hematocrit estimation was done [13, 14]. Weight, length and occipitofrontal circumference (OFC) were recorded as per standard technique. At 3 months (± 7 days) of age, hemoglobin and serum ferritin were estimated Hemoglobin/hematocrit was estimated by coulter method and ferritin was measured by two site immunoenzymatic assay. During follow up, iron supplementation, type of feeding, any illness requiring hospitalization and blood transfusion if any was recorded.

The primary outcome of our study was serum ferritin at 3 months of age. Secondary outcomes were polycythemia, need for partial exchange transfusion, hypoglycemia and neonatal hyperbilirubinemia requiring phototherapy. In addition other secondary outcomes were hemoglobin and anthropometric assessment at 3 months of age. Polycythemia was defined as venous hematocrit > 65% [15]. Partial exchange was done in asymptomatic infant with hematocrit > 70% and in symptomatic infants with hematocrit > 65% [15]. Symptomatic polycythemia was defined as hematocrit > 65% with any of the symptoms such as respiratory distress, apnea, cyanosis, seizures, necroterizing enterocolitis (NEC), hypoglycaemia, renal vein thrombosis and disseminated intravascular coagulation (DIC). Iron deficiency at 3 months was defined as serum ferritin < 50 ng/ml [16]. Management of hyperbilirubinemia was based on AAP guidelines [17].

### Sample size and statistical analysis

We could not find any study comparing ECC and DCC done exclusively in SGA infants. In a study by Geetanath et al. [18], the ferritin levels in term infants in early clamping group at 3 months of age were 39% of the cord ferritin levels. We hypothesized that in the DCC group, the serum ferritin levels at 3 months of age would be 64% of cord ferritin levels i.e. 25% more than early clamping group. We accounted for 10% attrition in follow-up and also for 10% of infants who would be erroneously labelled as SGA on antenatal USG but would turn out appropriate for gestational age (AGA) postnatally or require resuscitation. We calculated that 75 infants would be required in each group to detect the estimated difference with a power of 80% and two tailed alpha error of 0.05.

Analysis of data was done by using SPSS software version 21. Fisher exact or Chi square test was used to compare categorical variables. Student’s t test or Mann Whitney test were used to compare continuous variables as appropriate. Two sided *p* value of < 0.05 was considered significant.

## Results

**Fig. 1:**
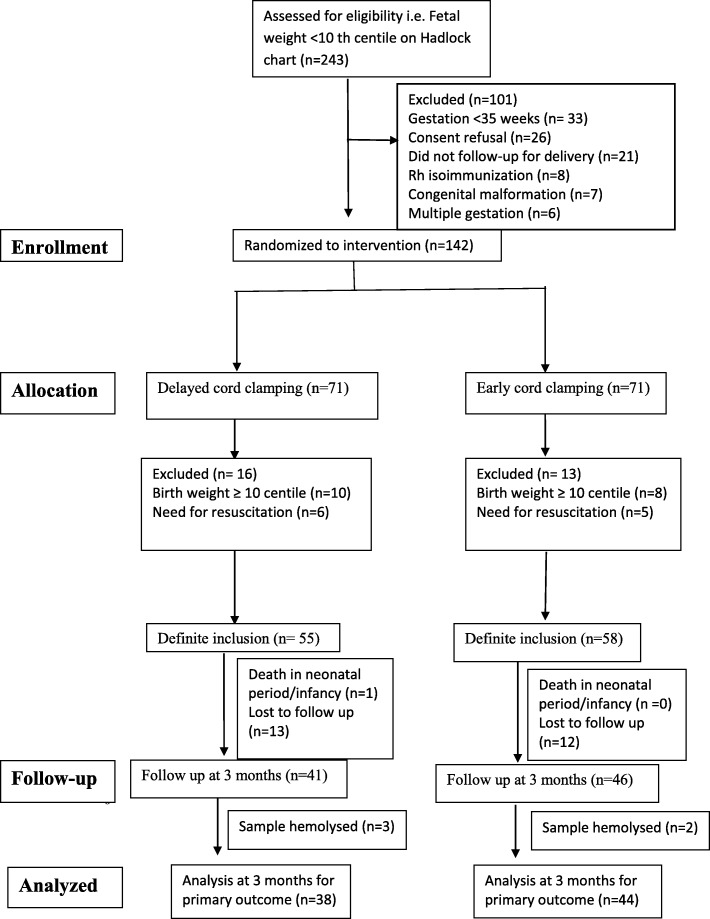
Consort flow diagram

A total of 2514 women underwent antenatal ultrasonography during the study period. Of these, 243 fetuses weighed less than 10th centile on Hadlock chart. Before delivery, 142 infants underwent randomization and subsequently 113 infants underwent definite inclusion. Forty four infants in ECC and 38 in DCC group were analyzed at 3 months for the primary outcome (Fig. 1).

The baseline maternal and neonatal characteristics were similar in both the groups except the time of cord clamping which differed by study design (Table 1).

**Table 1:** Baseline maternal and neonatal parameters

Parameters	DCC (*n* = 55)	ECC (*n* = 58)	*P* value
Maternal			
Prepregnancy weight (kg)	54.48 (8.1)	55.71 (11.6)	0.51
Weight gain during pregnancy (kg)	9.77 (1.9)	10.3 (1.5)	0.09
Height (cm)	155.14 (4.6)	155.33 (5.1)	0.83
BMI (Kg/m^2^)	22.48 (3.2)	23.02 (4.3)	0.44
Hemoglobin (g/dl)	11.73 (1.1)	11.52 (1.3)	0.36
Cesarean section^a^	43 (78.2)	40 (67.3)	0.19
Time of cord clamping (seconds)	62.6 (6.5)	12.05 (3.7)	<.001
Neonatal			
Gestational age (weeks)	37.49 (1.5)	37.72 (1.5)	0.41
Male^a^	23 (41.8)	33 (56.9)	0.10
Birth weight (g)	2188 (334)	2202 (389)	0.84
Length (cm)	45.97 (3.2)	46.16 (3.3)	0.75
OFC (cm)	32.23 (1.6)	32.55 (2.0)	0.36
Cord Ferritin (ng/ml)^b^	108 (48–212)	125 (51–193)	0.78

Mean (SD) unless stated otherwise, ^a^n(%), ^b^Median (IQR). Abbreviations: *BMI* Body mass index, *OFC* Occipito frontal circumference

At 3 months of age, the serum ferritin levels were higher in DCC group. Fewer infants at 3 months of age developed iron deficiency in DCC group (Table 2) [NNT being 4; 95% CI (2–25)]. The proportion of infants who developed polycythemia was significantly higher in DCC group. However there was no difference in proportion of infants who developed symptomatic polycythemia or required partial exchange transfusion. There were no other differences in hematological and clinical outcomes in neonatal period (Table 3). Hemoglobin (Table 2) and growth parameters at 3 months were also similar between the groups (Table 4).

**Table 2:** Hematological parameters at 3 months

Parameters	DCC (*n* = 38)	ECC (*n* = 44)	Relative risk or Difference (95%CI)	*P* value
Ferritin (ng/ml)^b^	86 (43.35–134.75)	50.5 (29.5–83.5)		0.01
Ferritin< 50 ng/ml^a^	9 (23.6)	21 (47.7)	0.4692 (0.25 to 0.94)	0.03
Hemoglobin (g/dl)	10.18 (0.8)	10.37 (1.4)	0.19 (−0.32 to 0.70)	0.45

Mean (SD) unless stated otherwise, ^a^n(%), ^b^median (IQR)

**Table 3:** Hematological and clinical outcomes in neonatal period

Parameters	DCC (*n* = 55)	ECC (*n* = 58)	*P* value
Hematocrit at 2 h (%)^a^	63.13 (6.3)	61.15 (5.3)	0.07
Polycythemia (Hct > 65%)	23 (41.8)	12 (20.6)	0.01
Hematocrit > 70%	4 (7.2)	3 (5.1)	0.71
Symptomatic polycythemia	2 (3.6)	1 (1.7)	0.52
Partial exchange	6 (10.9)	3 (5.1)	0.31
Respiratory distress	3 (5.4)	2 (3.4)	0.60
Hypoglycemia	6 (10.9)	8 (13.8)	0.64
Peak bilirubin (mg/dl)^a^	13.75 (1.8)	15.4 (3.5)	0.23
Duration of phototherapy (hrs)^a^	30 (16.9)	31.5 (14.6)	0.85
NEC	1 (1.8)	1 (1.8)	0.97
DIC	1 (1.8)	0	0.48
RVT	0	0	1.0
Mortality	1 (1.8)	0	0.48

N (%) except ^a^Mean (SD). Abbreviations- *NEC* Necrotizing enterocolitis, *RVT* Renal vein thrombosis, *DIC* Disseminated intravascular coagulation

**Table 4:** Anthropometric parameters/type of feeding and other outcomes at 3 months

Parameter	DCC (*n* = 38)	ECC (*n* = 44)	*P* value
Weight (g)	4683 (738)	4867 (681)	0.24
Length (cm)	54.68 (3.3)	55.96 (3.8)	0.11
OFC (cm)	37.73 (1.4)	38.10 (1.9)	0.32
Exclusive breast feeding^a^	20 (52.6)	21 (47.7)	0.65
Exclusive Formula feeding^a^	7 (18.4)	7 (16)	0.76
Mixed feeding^a^	11 (28.9)	16 (36.4)	0.47
Iron supplementation	0	0	1.0
Blood transfusions	0	0	1.0
Any hospitalization	0	0	1.0

Mean (SD) except ^a^n (%). Abbreviation: *OFC* Occipito frontal circumference

## Discussion

The burden of SGA infants in India is high with nearly 47% of neonates being SGA [19]. Because of low iron stores at birth; these neonates are at increased risk of developing iron deficiency and anemia later [8, 9]. DCC is a well-established strategy to decrease the burden of iron deficiency anemia in infancy. However the role of this simple cost effective intervention has not been systematically evaluated in the vulnerable SGA population.

We found that infants in DCC group had significantly higher serum ferritin levels compared to ECC group. Our findings of increased iron stores are similar to those observed in other studies [20–22]. Chaparo et al. in a study randomized 476 term AGA infants to DCC (at 180 s) and ECC groups [20]. Serum ferritin levels were significantly higher at 6 months in DCC group. Similarly Gupta et al. studied 102 term infants born to anemic mothers (Hb < 10 g/dl) and observed higher ferritin levels in DCC group at 3 months [21]. However these studies did not report outcomes specifically for SGA infants. Delaying cord clamping at birth could possibly have led to successful placental transfusion resulting in improved ferritin levels.

The proportion of infants with iron deficiency at 3 months of age in our study was lower in DCC group although hemoglobin levels were similar. We did not observe difference in hemoglobin probably because iron deficiency progresses in stages with erythropoiesis affected only after bone marrow iron stores and serum ferritin levels have decreased considerably [23]. A longer follow up might have possibly detected the difference in hemoglobin levels. A recent Cochrane review comparing early and delayed cord clamping strategies in term infants has also shown similar results of lower incidence of iron deficiency in DCC group and similar hemoglobin in both the groups [6].

Secondary outcomes of our study included hemoglobin and hematocrit at 2 h, polycythemia and other clinical outcomes in the neonatal period. We found increased hemoglobin and hematocrit at 2 h in DCC group, although it did not reach statistical significance. The incidence of polycythemia was higher in DCC group which is contrary to the findings of Cochrane systematic review on the effect of DCC in term infants [6]. This could possibly be due to increased baseline risk of polycythemia in SGA infants compared to AGA infants (15% vs 2%) [24]. DCC by providing additional RBCs increased the risk of polycythemia in these infants. However we did not find any difference in the rates of symptomatic polycythemia or partial exchange transfusions in the two groups. Similarly no difference in partial exchange transfusions were noted in two other studies comparing DCC and ECC on term AGA infants [25, 26].

Hyperbilirubinemia is another complication that may be related to polycythemia and delayed cord clamping. We found no significant difference in the proportion of infants having significant hyperbilirubinemia requiring phototherapy. Peak bilirubin levels and duration of phototherapy were also similar. These findings are in contrast to the results of studies conducted on term infants in which fewer infants in the ECC group required phototherapy for hyperbilirubinemia than in the DCC group [6]. This could be due to accelerated hepatic maturation in SGA infants [27]. Other clinical outcomes such as hypoglycemia, respiratory distress, necrotizing enterocolitis, renal vein thrombosis, disseminated intravascular coagulation and mortality were similar. There were no differences in the anthropometric measures (weight, length & OFC), feeding patterns, blood transfusions and hospitalizations between the groups at 3 months of age.

This is the first randomized controlled trial conducted exclusively on SGA infants comparing the effect of delayed and early cord clamping on early neonatal outcomes and iron stores at 3 months of age. The findings could have important implication in developing countries where a large proportion of infants delivered are SGA and are at high risk of developing iron deficiency.

Our study had few limitations*.* Iron stores and hematological parameters were not evaluated beyond 3 months of age which could have provided important insight during follow up of these infants. We randomized 142 infants, however due to higher number of post randomization exclusions, attrition and unexpected hemolysis of samples; we could not reach the expected sample size for the primary outcome. Further enrolment was not feasible as the trial had to be stopped due to logistic issues. Another limitation of our study is that we did not follow-up those randomized infants whose weight was at or above 10th centile and those who needed resuscitation at birth; therefore it was not possible for us to perform intention to treat analysis.

## Conclusions

DCC leads to improved iron stores in SGA infants ≥35 weeks at 3 months of age without increasing the risk of symptomatic polycythemia, need for partial exchange transfusions or morbidities associated with polycythemia.

## Abbreviations

AGA: Appropriate for gestational age
DCC: Delayed cord clamping
DIC: Disseminated intravascular coagulation
ECC: Early cord clamping
NEC: Necrotizing enterocolitis
OFC: Occipito frontal circumference
RVT: Renal vein thrombosis
SGA: Small for gestational age
